# Exploring foundation doctors’ self-reported confidence in the assessment and management of mental health conditions

**DOI:** 10.1192/bjb.2023.48

**Published:** 2024-04

**Authors:** George Gillett, Owen Davis, Amarit Gill, Clare van Hamel

**Affiliations:** 1 Institute of Psychiatry, Psychology and Neuroscience, King's College London, London, UK; 2 UK Foundation Programme Office, Birmingham, UK; 3 Severn Postgraduate Medical Education Foundation School, Bristol, UK

**Keywords:** Clinical governance, education and training, consent and capacity, ethics

## Abstract

**Aims and method:**

This study assesses newly qualified doctors’ confidence in practising clinical skills related to the assessment and management of mental health conditions and how this correlates with other areas of medicine. We conducted a national survey of 1311 Foundation Year 1 doctors in the UK. Survey items assessed confidence recognising mentally unwell patients, conducting a mental state examination, assessing cognition and mental capacity, formulating a psychiatric diagnosis and prescribing psychotropic medications.

**Results:**

A substantial proportion of surveyed doctors lacked confidence in their clinical skills related to mental health and prescribing psychotropic medications. Network analysis revealed that items corresponding to mental health were highly correlated, suggesting a potential generalised lack of confidence in mental healthcare.

**Clinical implications:**

We identify areas of lack of confidence in some newly qualified doctors’ ability to assess and manage mental health conditions. Future research might explore how greater exposure to psychiatry, integrated teaching and clinical simulation might better support medical students for future clinical work.

Medical graduates work across a plethora of clinical placements during their training as junior doctors, before specialising in different specialties.^1^ Given that many doctors do not work in a psychiatry placement during their postgraduate training, medical school represents a unique and important opportunity to teach clinical skills pertaining to psychiatry, which remain relevant regardless of doctors’ eventual specialty.^1^ In the UK, this is recognised by the Foundation Programme curriculum, which expects Foundation Year doctors to be competent in ‘recognition and assessment’ of various mental health conditions, ‘recognising the need for urgent intervention to treat mental health problems’, ‘assessing capacity’, ‘the mental state examination’, ‘managing a disturbed or challenging patient’ and familiarity with ‘common psychotropic medications’, among other clinical skills.^2^ The importance of equipping clinicians, across all specialties, with the basic skills and knowledge to treat psychiatric needs has been highlighted by the Royal College of Psychiatrists and the General Medical Council.^3,4^

However, the development of clinical competence in psychiatry may harbour unique challenges and require specialist attention in medical school curricula.^5^ For example, literature suggests that students may find aspects of psychiatric assessment, such as the mental state examination and assessment of cognition, to be more challenging compared with other aspects of their training.^6,7^ Furthermore, clinicians working in specialties other than psychiatry report difficulties with clinical skills such as the assessment of cognition and mental capacity, the mental state examination and the prescribing of psychotropic medications.^8–10^ It is therefore possible that clinical skills related to psychiatry represent areas of relatively unmet need in medical school curricula and clinical practice.

Here, we present an analysis of a large-scale survey of newly qualified doctors in the UK to assess their self-reported confidence in assessing and managing mental health conditions. Survey items pertained to the assessment and management of both mental and physical health conditions, as relevant to competencies outlined in the UK's Foundation Programme curriculum.^2^ Topics included recognising acute mental illness, conducting a mental state examination, assessing cognition and mental capacity, formulating psychiatric diagnoses and prescribing psychotropic medications. We summarise respondents’ confidence across these domains and contextualise them against other clinical skills. Finally, we perform a network analysis to assess how doctors’ self-reported confidence in assessing and managing mental health conditions correlates with other clinical skills.

## Method

### Study design

Anonymous online surveys were cascaded to newly qualified Foundation Year 1 doctors in the UK during August 2021, their first month of work. Questions included demographics (gender, age, ethnicity and medical school) and Likert-scale questions about respondents’ training and preparedness for clinical work. Items assessing respondents’ self-reported confidence in the assessment and management of mental health conditions included: ‘To what extent do you agree with the following statements? (a) I feel confident in recognising the acutely mentally unwell patient, (b) I feel confident in formulating mental health diagnoses, (c) I feel confident in conducting a mental state examination, (d) I feel confident in assessing cognition, (e) I feel confident in performing a capacity assessment’ and ‘I feel confident in prescribing the following drugs: (f) antidepressants, (g) anti-anxiety medications, (h) antipsychotics and (i) medications for agitation and delirium'. For context, respondents’ self-reported confidence in the assessment of physical health conditions was assessed with the following items: ‘To what extent do you agree with the following statements? (a) I feel confident in recognising the acutely physically unwell patient, (b) I feel confident in formulating physical health diagnoses, (c) I feel confident performing practical procedures’ and ‘I feel confident in prescribing the following drugs: (d) simple analgesics, (e) narcotic analgesics, (f) bronchodilators, (g) inhaled steroids, (h) antimicrobials, (i) anticoagulants, (j) insulin, (k) oral antidiabetic drugs and (l) intravenous fluids'. Respondents rated their responses to each item: ‘strongly agree’, ‘agree’, ‘neutral’, ‘disagree’ or ‘strongly disagree’. The sample size was limited to respondents who answered all survey items relevant to this analysis, with the exception of demographic questions.

### Statistical analysis

All statistical analyses were defined *a priori.* For categorical analyses, the proportion of respondents agreeing (sum of ‘agree’ and ‘strongly agree’) and disagreeing (sum of ‘disagree’ and ‘strongly disagree’) with each statement was calculated and 95% confidence intervals derived. To compare mental health items with contextual physical health items, McNemar chi-squared tests were performed for paired categorical data for each relevant comparison. For further analyses, Likert responses were coded from 1 (‘strongly disagree’) to 5 (‘strongly agree’), mean score and 95% confidence intervals were derived and non-parametric tests for ordinal data were performed. Friedman tests were used to identify differences across multiple items, and effect sizes were calculated using Kendall's coefficient of concordance (*W*). Wilcoxon signed-rank tests were then performed to compare relevant items and Wilcoxon effect sizes (*r*) were calculated. The Benjamini–Hochberg procedure was used to adjust for multiple comparisons. Finally, we performed an association network analysis to demonstrate the strength of correlations across all items. In an association network, the edge weights and proximity of nodes to other nodes represent the strength of correlation between survey items. In this analysis, correlation between each survey item was assessed using polychoric correlations for ordinal data. All analyses were conducted using R version 4.0.0 for Mac; *P*-values below 0.05 were considered statistically significant.

### Ethics statement

Ethical approval was not required as the project was considered to be a service evaluation project of anonymised data. All survey respondents voluntarily consented to participation in the project.

## Results

### Sample demographics

In total, 1311 respondents answered all survey items relevant to this analysis, representing 17.0% of the total cohort of Foundation Year 1 doctors matched to a placement due to start in August 2021. The sample comprised graduates from all UK medical schools. Regarding gender, of respondents providing relevant information, 693 (60.1%) were female, 456 (39.5%) were male and 4 (0.3%) were non-binary. Regarding ethnicity, 648 (58.7%) were White, 313 (28.4%) were Asian or Asian British, 45 (4.1%) were Black or Black British and 98 (8.9%) reported their ethnicity as ‘Other’. Regarding age, 778 (66.4%) were aged 21–25 years, 305 (26.0%) aged 26–30 years, 59 (5.0%) aged 31–35 years and 30 (2.6%) aged 36 years or older.

### Confidence assessing mental health conditions

**Fig. 1:**
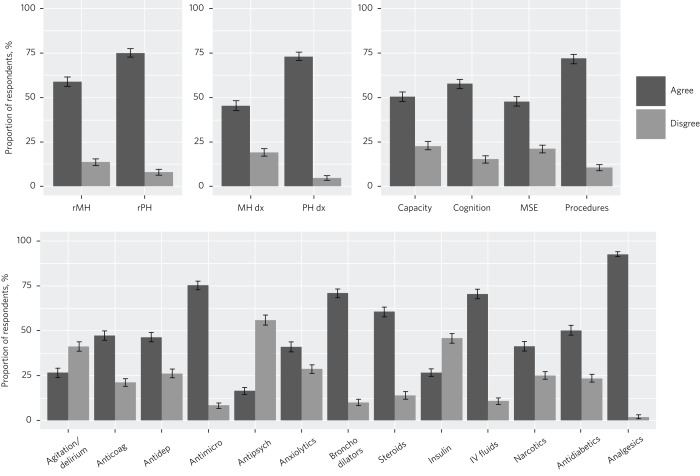
Respondents’ confidence with clinical skills and prescribing: categorical analysis showing the proportion of respondents agreeing or disagreeing that they feel confident with various clinical skills and prescribing. Clinical skills: rMH, recognising the mentally unwell patient; rPH, recognising the physically unwell patient; MH dx, formulating a mental health diagnosis; PH dx, formulating a physical health diagnosis; MSE, mental state examination. Prescribing: Agitation/delirium, medications for agitation and delirium; Anticoag, anticoagulants; Antidep, antidepressants; Antimicro, antimicrobials; Antipsych, antipsychotics; Steroids, inhaled steroids; Narcotics, narcotic analgesics; IV, intravenous; Antidiabetics, per oral antidiabetics; Analgesics, simple analgesics. Error bars represent 95% confidence intervals.

The majority of doctors reported feeling confident recognising the acutely mentally unwell patient (58.9%, 95% CI 56.2–61.6), assessing cognition (57.7%, 95% CI 54.5–60.3) and assessing capacity (50.4%, 95% CI 47.7–53.1), and a substantial minority reported feeling confident conducting a mental state examination (MSE) (47.8%, 95% CI 45.1–50.5) and formulating mental health diagnoses (45.3%, 95% CI 42.6–48.0) (Fig. 1). Nonetheless, a substantial minority of doctors disagreed with feeling confident in each of those domains (capacity: 22.7%, 95% CI 20.4–24.9; MSE: 21.1%, 95% CI 18.9–23.3; formulating mental health diagnoses: 19.1%, 95% CI 16.9–21.2; cognition: 15.3%, 95% CI 13.3–17.2; recognising the acutely mentally unwell patient: 13.6%, 95% CI 11.7–21.2) (Fig. 1).

When contextualised against comparator physical health items, doctors were more likely to report feeling confident recognising acutely physically unwell patients compared with recognising acutely mentally unwell patients (75.1%, 95% CI 72.8–77.5 *v*. 58.9%, 95% CI 56.2–61.6; McNemar's χ^2^ = 129.9, *P* < 0.001) and more likely to feel confident formulating physical health diagnoses compared with mental health diagnoses (73.0%, 95% CI 70.6–75.4 *v*. 45.31%, 95% CI 42.6–48.0; McNemar's χ^2^ = 34.1, *P* < 0.001). Doctors were more likely to report feeling confident performing practical procedures (71.9%, 95% CI 69.5–74.4) compared with conducting an MSE (47.8%, 95% CI 45.1–50.5; McNemar's χ^2^ = 40.6, *P* < 0.001), assessing cognition (57.7%, 95% CI 54.5–60.3; McNemar's χ^2^ = 100.0, *P* < 0.001) or assessing capacity (50.4%, 95% CI 47.7–53.1; McNemar's χ^2^ = 53.5, *P* < 0.001).

**Fig. 2:**
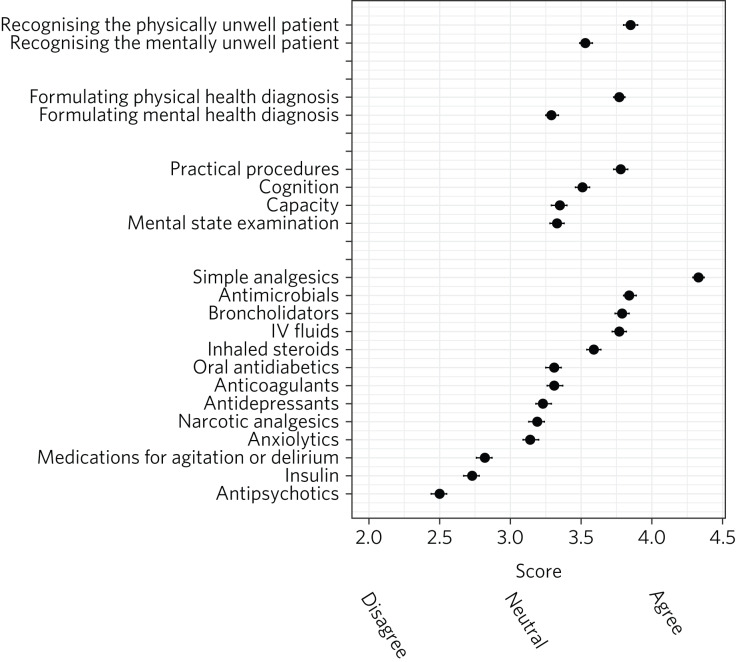
Respondents’ confidence with clinical skills and prescribing: ordinal analysis presenting mean scores on a Likert scale coded from 1 (‘strongly disagree’) to 5 (‘strongly agree’), with 95% confidence intervals for each item.

Figure 2 presents a plot of mean scores for each item on a five-point Likert scale (1, ‘strongly disagree’; 5, ‘strongly agree’). When analysed as ordinal rather than categorical data, significant results were replicated. There were significant differences between self-reported confidence in recognising acutely physically unwell compared with mentally unwell patients (3.85, 95% CI 3.80–3.90 *v*. 3.53, 95% CI 3.49–3.58; *r* = 0.35, *P* < 0.001), formulating physical compared with mental health diagnoses (3.77, 95% CI 3.73–3.81 *v*. 3.29, 95% CI 3.25–3.34; *r* = 0.53, *P* < 0.001) and performing practical procedures (3.78, 95% CI 3.73–3.83) compared with conducting an MSE (3.33, 95% CI 3.28–3.38; *r* = 0.38, *P* < 0.001), assessing capacity (3.35, 95% CI 3.29–3.40; *r* = 0.36, *P* < 0.001) and assessing cognition (3.51, 95% CI 3.46–3.56; *r* = 0.275, *P* < 0.001).

### Confidence prescribing psychotropic medication

For prescribing psychotropic medication, a substantial minority of doctors reported feeling confident prescribing antidepressant (46.5%, 95% CI 43.8–49.2) or anxiolytic (41.2%, 95% CI 38.5–43.9) medications, but fewer reported feeling confident prescribing medications to manage agitation and delirium (26.7%, 95% CI 24.3–29.1) or antipsychotic medication (16.5%, 95% CI 14.5–18.5). The majority of doctors explicitly disagreed with feeling confident prescribing antipsychotic medication (55.9%, 95% CI 53.2–58.6) and a substantial minority did not feel confident prescribing other psychotropic medication (medications for agitation and delirium: 41.4%, 95% CI 38.8–44.1; anxiolytics: 28.6%, 95% CI 26.2–31.1; antidepressants: 26.2, 95% CI 23.9–28.6). Results for confidence prescribing various physical health medications are summarised in Supplementary Material 1, available at https://doi.org/10.1192/bjb.2023.48.

There was a general trend of doctors feeling more confident prescribing physical health medications compared with psychotropic medications, with the exception of insulin and narcotic analgesics (antipsychotics < insulin < medications for agitation and delirium < anxiolytics, narcotics, antidepressants < all other medications; *P* < 0.05; Fig. 2). Effect sizes for these comparisons are presented in Supplementary Material 2 and 3.

### Association between survey items

**Fig. 3:**
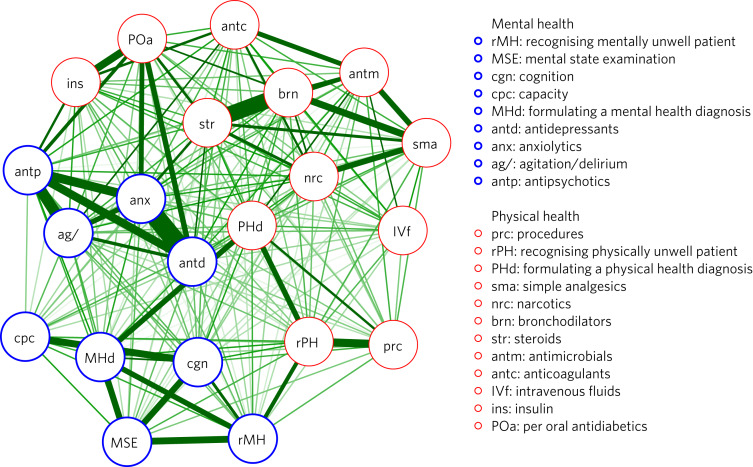
Association network analysis for participants’ responses to all items. Edge weights and position of nodes represent the strength of polychoric correlation with other items. Very weak correlations (*r* < 0.2) are excluded.

Figure 3 displays an association network of correlations between respondents’ self-reported confidence for all domains measured. The network suggests that items corresponding to mental health generally cluster together, perhaps across two separable clusters: items assessing confidence in clinical assessment (cognition, capacity, mental state examination, formulating mental health diagnoses and recognising the acutely mentally unwell patient) and items assessing confidence prescribing (antidepressants, anxiolytics, antipsychotics and medications for agitation/delirium). This may suggest the existence of an underlying factor uniting the domains related to mental health. This finding may suggest that our above-mentioned results reflect a generalised and related lack of confidence in assessing and managing mental health conditions across multiple domains, rather than the existence of multiple idiosyncratic discrepancies specific to each comparison. Correlation coefficients for all items are presented in Supplementary Material 4.

## Discussion

Our findings identify that although the majority of newly qualified doctors in our survey report feeling confident in assessing and managing mental health conditions, a substantial minority lacked confidence in key areas. In particular, as many as one in five respondents lacked confidence in conducting a mental state examination and assessing mental capacity, clinical skills that are often required in Foundation Doctor placements and that are highly relevant to clinical specialties beyond psychiatry. When contextualised against items related to physical health conditions, doctors generally reported feeling less confident with clinical skills related to mental health.

For prescribing, we identified a similar pattern. Although initiating specialist psychotropic medication might be beyond the competencies expected of a Foundation Doctor, familiarity with prescribing such medication is likely to be relevant to doctors’ roles in various specialties, given the community prevalence of psychotropic medication prescriptions. For instance, Foundation Doctors frequently prescribe psychotropic medication when continuing in-patients’ regular pre-admission medication, and a knowledge of withdrawal effects, need for re-titration where appropriate and the adverse effects associated with such medication is likely to be relevant to doctors working in a number of specialties. It is therefore salient, for example, that newly qualified doctors report feeling significantly less confident in prescribing antidepressants compared with oral antidiabetic medication, despite antidepressant prescriptions exceeding the number of antidiabetic prescriptions in the UK.^11^ Likewise, respondents’ lack of confidence in prescribing for agitation and delirium is concerning, given that delirium is a common presentation among general hospital in-patients, scenarios often arise out of hours and mismanagement can have significant consequences.^12^

Interestingly, our network analysis revealed that confidence in clinical skills related to mental health closely correlated across respondents, which may reflect an underlying generalised lack of confidence in assessing and managing mental health conditions. An improved understanding of the factors determining doctors’ confidence to assess and manage mental health conditions, and particularly how it can be enhanced, is likely to be important in better equipping doctors to meet patients’ psychiatric needs. A number of proposed interventions have promise and would be worthy of future research, including the use of clinical scenarios with simulated patients,^13^ the use of teaching aids such as videos,^14,15^ greater exposure to psychiatry in the medical school curriculum,^16,17^ clinical case team-based learning^18^ and delivering psychiatry teaching in integrated and liaison clinical settings.^19,20^ However, findings relating to the effectiveness of many of these interventions have been inconsistent to date.^21,22^

This study exhibits limitations. The survey features self-reported, rather than objective, measures of clinical confidence. Although it was necessary to use self-reported measures in our national study, future research might focus on validating objective or patient-reported measures of doctors’ competence in assessing and treating mental health conditions. It is also possible that the method of distribution of the survey (cascading it via emails and circulars) introduced bias in our study population, given the survey's response rate of 17.0%. The survey nevertheless benefitted from a substantial sample size, with responses covering all medical and foundation schools in the UK, and a diverse demographic population, representing the largest investigation into newly qualified doctors’ preparedness for clinical practice to our knowledge. Despite this, further research to confirm these findings using diverse populations and study methodology is encouraged.

The cross-sectional nature of the survey meant that it was not possible to assess for individual changes in doctors’ self-reported confidence, and future research might investigate the effects of foundation and specialist clinical training on such outcomes. In particular, the effect of the Foundation Programme on these outcomes might be explored, given that a number of doctors undertake clinical placements in psychiatry.^18^ Finally, it was not possible to assess clinical skills related to risk assessment, child and adolescent psychiatry and substance misuse, which have been identified as potential areas of difficulty for clinicians and would be a worthy focus of future research.^10^

## Supporting information

Gillett et al. supplementary material Gillett et al. supplementary material

## Data Availability

The data that support the findings of this study are available from the corresponding author, G.G., on reasonable request.

## About the authors

**George Gillett** is an NIHR Academic Clinical Fellow at the Institute of Psychiatry, Psychology and Neuroscience, King's College London, UK. **Owen Davis** is a UK Foundation Programme Leadership Fellow in the UK Foundation Programme Office, Birmingham, UK; **Amarit Gill** is a UK Foundation Programme Leadership Fellow in the UK Foundation Programme Office, Birmingham, UK; **Clare van Hamel** is Foundation School Director and Associate Post Graduate Dean of Severn Postgraduate Medical Education Foundation School, Bristol, UK.
