# Supplementary material for: Exploring foundation doctors’ self-reported confidence in the assessment and management of mental health conditions
Source: BJPsych Bull. 2024 Apr;48(2):139–44. doi: 10.1192/bjb.2023.48 (PMC10985717; doi:10.1192/bjb.2023.48)
Supplement: Gillett et al. supplementary material [file S2056469423000487sup001.docx]

**Supplementary Material**

Exploring foundation doctors’ self-reported confidence in the assessment and management of mental health conditions

George Gillett^1*^, Owen Davis^2^, Amarit Gill^2^, Clare van Hamel^3^

^1^NIHR Academic Clinical Fellow, Institute of Psychiatry, Psychology and Neuroscience, King’s College London, London, UK. ORCID ID: 0000-0002-0270-9369

^2^UK Foundation Programme Leadership Fellow, UK Foundation Programme Office, UK

^3^Severn Foundation School Director & Associate Post Graduate Dean, UK

*** Correspondence:**Dr George Gillett
george.1.gillett@kcl.ac.uk

**Supplementary Material 1: Summary statistics of participants’ responses**

N = 1,311 respondents.

|  |  | **Agree [95% CI]** | **Disagree [95% CI]** | **Median (LQ, UQ)** | **Mean, [95% CI]** |
| --- | --- | --- | --- | --- | --- |
| **Clinical items** | **MSE** | 47.75  [45.05, 50.45] | 21.13  [18.92, 23.34] | 3 (3,4) | 3.33 [3.28, 3.38] |
|  | **Cognition** | 57.67  [54.99, 60.34] | 15.26  [13.31, 17.20] | 4 (3,4) | 3.51 [3.46, 3.56] |
|  | **Capacity** | 50.42  [47.71, 53.13] | 22.65  [20.39, 24.92] | 4 (3, 4) | 3.35 [3.29, 3.40] |
|  | **Recognising Mentally Unwell Patient** | 58.89  [56.22, 61.55] | 13.58  [11.72, 15.43] | 4 (3,4) | 3.53 [3.49, 3.58] |
|  | **Formulating Mental Health Diagnosis** | 45.31  [42.61, 48.00] | 19.07  [16.94, 21.20] | 3 (3,4) | 3.29 [3.25, 3.34] |
|  | **Recognising Physically Unwell Patient** | 75.13  [72.79, 77.47] | 7.93  [6.47, 9.40] | 4 (4,4) | 3.85 [3.80, 3.90] |
|  | **Formulating Physical Health Diagnosis** | 73.0  [70.59, 75.40] | 4.65  [3.51, 5.79] | 4 (3, 4) | 3.77 [3.73, 3.81] |
|  | **Practical Procedures** | 71.93  [69.50, 74.36] | 10.6  [8.94, 12.27] | 4 (3,4) | 3.78 [3.73, 3.83] |
| **Prescribing** | **Antidepressants** | 46.53  [43.83, 49.23] | 26.24  [23.86, 28.62] | 3 (2,4) | 3.23 [3.18, 3.29] |
|  | **Anti-anxiety medication** | 41.19  [38.53, 43.85] | 28.6  [26.16, 31.05] | 3 (2,4) | 3.14 [3.09, 3.20] |
|  | **Antipsychotics** | 16.48  [14.47, 18.48] | 55.91  [53.22, 58.60] | 2 (2,3) | 2.50 [2.44, 2.55] |
|  | **Medications for agitation/delirium** | 26.7  [24.3, 29.09] | 41.42  [38.75, 44.09] | 3 (2,4) | 2.82 [2.76, 2.87] |
|  | **Anticoagulants** | 47.29  [44.59, 49.99] | 21.28  [19.07, 23.50] | 3 (3,4) | 3.31 [3.26, 3.37] |
|  | **Antimicrobials** | 75.44  [73.11, 77.77] | 8.39  [6.89, 9.89] | 4 (4,4) | 3.84 [3.80, 3.89] |
|  | **Bronchodilators** | 71.09  [68.64, 73.54] | 10.07  [8.44, 11.70] | 4 (3,4) | 3.79 [3.74, 3.84] |
|  | **Inhaled steroids** | 60.56  [57.92, 63.21] | 14.26  [12.37, 16.16] | 4 (3,4) | 3.59 [3.54, 3.64] |
|  | **Insulin** | 26.54  [24.15, 28.93] | 46.07  [43.37, 48.77] | 3 (2,4) | 2.73 [2.67, 2.78] |
|  | **Intravenous fluids** | 70.63  [68.17, 73.10] | 10.91  [9.22, 12.60] | 4 (3,4) | 3.77 [3.72, 3.82] |
|  | **Narcotic analgesics** | 41.34  [38.68, 44.01] | 25.1  [22.75, 27.44] | 3 (2,4) | 3.19 [3.13, 3.24] |
|  | **Oral anti-diabetics** | 50.34  [47.64, 53.05] | 23.49  [21.20, 25.79] | 4 (3,4) | 3.31 [3.25, 3.36] |
|  | **Simple analgesia** | 92.68  [91.27, 94.09] | 2.06  [1.29, 2.83] | 4 (4,5) | 4.33 [4.29, 4.37] |

**Supplementary Material 2: Comparison between clinical skill items; effect sizes**

N = 1,311 respondents. Friedman χ2 = 292.21, p < 0.001, Kendall’s W = 0.07. Wilcoxon Signed-Rank tests performed to compare individual items and derive effect size. Correction for multiple comparisons made using Benjamini-Hochberg procedure. All p values are <0.001 except where stated. n.s. denotes non-significant (p>0.05).

|  |  | **Recognising physical illness** | **Formulating PH diagnosis** | **Practical Procedures** | **Cognition** | **Capacity** |
| --- | --- | --- | --- | --- | --- | --- |
| **Clinical skills** | **Recognising mentally unwell** | 0.351 | - | - | - | - |
|  | **Formulating MH diagnosis** | - | 0.526 | - | - | - |
|  | **MSE** | - | - | 0.383 | 0.189 | n.s. |
|  | **Cognition** | - | - | 0.275 | - | 0.162 |
|  | **Capacity** | - | - | 0.363 | - | - |

**Supplementary Material 3: Comparison between prescribing items; effect sizes**

N = 1,311 respondents. Friedman χ2 = 5250.7, p < 0.001, Kendall’s W = 0.34. Wilcoxon Signed-Rank tests performed to compare individual items and derive effect size. Correction for multiple comparisons made using Benjamini-Hochberg procedure. All p values are <0.001 except where stated. n.s. denotes non-significant (p>0.05).

|  | **Antidepressants** | **Anxiolytics** | **Antipsychotics** | **For agitation/delirium** | **Anticoagulants** | **Antimicrobials** | **Bronchodilators** | **Inhaled steroids** | **Insulin** | **Intravenous fluids** | **Narcotics** | **Oral anti-diabetics** | **Simple analgesics** |
| --- | --- | --- | --- | --- | --- | --- | --- | --- | --- | --- | --- | --- | --- |
| **Antidepressants** | - | 0.174 | 0.646 | 0.373 | 0.067 (p=0.03) | 0.514 | 0.480 | 0.319 | 0.380 | 0.418 | n.s. | 0.095 | 0.763 |
| **Anxiolytics** | - | - | 0.602 | 0.314 | 0.148 | 0.570 | 0.548 | 0.407 | 0.323 | 0.487 | n.s. | 0.181 | 0.787 |
| **Antipsychotics** | - | - | - | 0.375 | 0.623 | 0.784 | 0.787 | 0.743 | 0.223 | 0.757 | 0.554 | 0.639 | 0.856 |
| **For agitation/delirium** | - | - | - | - | 0.423 | 0.711 | 0.683 | 0.599 | 0.074 (p=0.02) | 0.665 | 0.332 | 0.406 | 0.832 |
| **Anticoagulants** | - | - | - | - | - | 0.512 | 0.428 | 0255 | 0.485 | 0.416 | 0.118 | n.s. | 0.758 |
| **Antimicrobials** | - | - | - | - | - | - | n.s. | 0.252 | 0.731 | n.s. | 0.559 | 0.446 | 0.545 |
| **Bronchodilators** | - | - | - | - | - | - | - | 0.334 | 0.705 | n.s. | 0.523 | 0.418 | 0.583 |
| **Inhaled steroids** | - | - | - | - | - | - | - | - | 0.628 | 0.179 | 0.374 | 0.253 | 0.672 |
| **Insulin** | - | - | - | - | - | - | - | - | - | 0.700 | 0.386 | 0.535 | 0.839 |
| **Intravenous fluids** | - | - | - | - | - | - | - | - | - | - | 0.498 | 0.374 | 0.547 |
| **Narcotics** | - | - | - | - | - | - | - | - | - | - | - | 0.114 | 0.828 |
| **Oral anti-diabetics** | - | - | - | - | - | - | - | - | - | - | - | - | 0.742 |
| **Simple analgesia** | - | - | - | - | - | - | - | - | - | - | - | - | - |

**Supplementary Material 4: Correlation between all survey items**

N = 1,311 respondents. Polychoric correlation coefficients between all survey items. rMH: recognising the mentally unwell patient, rPH: recognising the physically unwell patient, MH dx: formulating mental health diagnosis, PH dx: formulating physical health diagnosis, Agitation/Delirium: medications for agitation & delirium, Anticoag: anticoagulants, Antidep: antidepressants, Antimicro: antimicorbials, Antipsych: antipsychotics, Steroids: inhaled steroids, Narcotics: narcotic analgesics, Antidiabetics: oral antidiabetics, Analgesics: simple analgesics.

|  | rPH | rMH | MSE | Cognition | Capacity | PH dx | MH dx | Analgesics | Narcotics | Bronchodilators | Steroids | Antimicro | Anticoag | IV fluids | Insulin | Antidiabetes | Antidep | Anxiolytics | Agitation/Delirium | Antipsych |
| --- | --- | --- | --- | --- | --- | --- | --- | --- | --- | --- | --- | --- | --- | --- | --- | --- | --- | --- | --- | --- |
| Procedures | 0.64 | 0.38 | 0.34 | 0.41 | 0.36 | 0.49 | 0.32 | 0.39 | 0.35 | 0.35 | 0.34 | 0.41 | 0.31 | 0.34 | 0.25 | 0.25 | 0.33 | 0.33 | 0.28 | 0.23 |
| rPH | - | 0.53 | 0.4 | 0.45 | 0.42 | 0.56 | 0.45 | 0.39 | 0.36 | 0.39 | 0.37 | 0.38 | 0.34 | 0.38 | 0.33 | 0.26 | 0.34 | 0.33 | 0.35 | 0.26 |
| rMH | - | - | 0.6 | 0.5 | 0.42 | 0.41 | 0.57 | 0.26 | 0.25 | 0.31 | 0.3 | 0.29 | 0.27 | 0.3 | 0.22 | 0.25 | 0.37 | 0.36 | 0.34 | 0.36 |
| MSE | - | - | - | 0.59 | 0.45 | 0.32 | 0.59 | 0.26 | 0.26 | 0.26 | 0.29 | 0.24 | 0.24 | 0.2 | 0.29 | 0.29 | 0.37 | 0.38 | 0.4 | 0.41 |
| Cognition | - | - | - | - | 0.61 | 0.44 | 0.47 | 0.27 | 0.31 | 0.36 | 0.38 | 0.32 | 0.33 | 0.28 | 0.32 | 0.3 | 0.36 | 0.36 | 0.38 | 0.38 |
| Capacity | - | - | - | - | - | 0.39 | 0.41 | 0.21 | 0.32 | 0.31 | 0.33 | 0.22 | 0.3 | 0.29 | 0.28 | 0.2 | 0.31 | 0.33 | 0.38 | 0.33 |
| PH dx | - | - | - | - | - | - | 0.57 | 0.41 | 0.42 | 0.42 | 0.45 | 0.47 | 0.39 | 0.41 | 0.38 | 0.36 | 0.37 | 0.37 | 0.36 | 0.31 |
| MH dx | - | - | - | - | - | - | - | 0.22 | 0.29 | 0.28 | 0.31 | 0.25 | 0.25 | 0.22 | 0.34 | 0.33 | 0.43 | 0.44 | 0.39 | 0.46 |
| Analgesics | - | - | - | - | - | - | - | - | 0.58 | 0.6 | 0.5 | 0.57 | 0.41 | 0.46 | 0.28 | 0.39 | 0.37 | 0.36 | 0.28 | 0.22 |
| Narcotics | - | - | - | - | - | - | - | - | - | 0.47 | 0.5 | 0.42 | 0.48 | 0.4 | 0.45 | 0.39 | 0.44 | 0.44 | 0.46 | 0.44 |
| Bronchodilators | - | - | - | - | - | - | - | - | - | - | 0.84 | 0.52 | 0.42 | 0.39 | 0.36 | 0.47 | 0.46 | 0.45 | 0.35 | 0.35 |
| Steroids | - | - | - | - | - | - | - | - | - | - | - | 0.49 | 0.44 | 0.42 | 0.41 | 0.49 | 0.46 | 0.49 | 0.42 | 0.42 |
| Antimicro | - | - | - | - | - | - | - | - | - | - | - | - | 0.55 | 0.46 | 0.35 | 0.4 | 0.44 | 0.41 | 0.34 | 0.28 |
| Anticoag | - | - | - | - | - | - | - | - | - | - | - | - | - | 0.46 | 0.49 | 0.42 | 0.4 | 0.4 | 0.47 | 0.44 |
| IV fluids | - | - | - | - | - | - | - | - | - | - | - | - | - | - | 0.4 | 0.33 | 0.31 | 0.32 | 0.36 | 0.3 |
| Insulin | - | - | - | - | - | - | - | - | - | - | - | - | - | - | - | 0.62 | 0.37 | 0.38 | 0.44 | 0.49 |
| Antidiabetes | - | - | - | - | - | - | - | - | - | - | - | - | - | - | - | - | 0.57 | 0.55 | 0.41 | 0.52 |
| Antidep | - | - | - | - | - | - | - | - | - | - | - | - | - | - | - | - | - | 0.92 | 0.51 | 0.64 |
| Anxiolytics | - | - | - | - | - | - | - | - | - | - | - | - | - | - | - | - | - | - | 0.56 | 0.66 |
| Agitation/Delirium | - | - | - | - | - | - | - | - | - | - | - | - | - | - | - | - | - | - | - | 0.71 |
